# Seroprevalence, Dual Seropositivity, and Associated Risk Factors of BVDV and BoHV-1 in Dual-Purpose Cattle from the Colombian Eastern Plains

**DOI:** 10.3390/v18070723

**Published:** 2026-06-30

**Authors:** Emerson Sánchez Carvajal, Jorge Luis Parra Arango, Karl Ciuoderis, Agustín Góngora Orjuela

**Affiliations:** 1 School of Animal Sciences, Research Group in Animal Reproduction and Genetics, Universidad de los Llanos, Villavicencio 500004, Colombia; agropecuariasgenes@hotmail.com (E.S.C.); jlparra@unillanos.edu.co (J.L.P.A.); agongora@unillanos.edu.co (A.G.O.); 2 Corporacion Corpotropica, Veterinary Research, Villavicencio 500001, Colombia

**Keywords:** bovine viral diarrhea virus (BVDV), Bovine herpesvirus 1 (BoHV-1), seroprevalence, dual seropositivity, cattle, epidemiology, risk factors, dual-purpose systems, livestock diseases

## Abstract

Bovine viral diarrhea virus (BVDV) and bovine herpesvirus type 1 (BoHV-1) are major pathogens affecting cattle worldwide, leading to significant economic losses in livestock production systems. This study aimed to estimate the seroprevalence and dual seropositivity of both viruses in dual-purpose cattle in Villavicencio, Colombia. A total of 1000 serum samples were collected from cattle across 30 farms and analyzed using ELISA. Seroprevalence for BVDV ranged from 0.0% to 78.7%, while BoHV-1 ranged from 0.0% to 100.0%. Dual seropositivity with both viruses was observed at the farm level, ranging from 0.0% to 52.2%. At the herd level, 26.7% (8/30) of the farms were seronegative for BVDV (95% CI: 14.2–44.5%), whereas only 3.3% (1/30) were seronegative for BoHV-1 (95% CI: 0.6–16.7%). Additionally, seven farms (23.3%) were simultaneously seronegative for both BVDV and BoHV-1. Overall, the findings demonstrate widespread exposure and non-random concurrent seropositivity of both viruses, highlighting the importance of herd management, biosecurity, and animal movement in shaping infection dynamics in dual-purpose cattle systems. Management practices, animal movement, and environmental factors were significantly associated with seropositivity.

## 1. Introduction

Bovine viral diarrhea virus (BVDV) and bovine herpesvirus type 1 (BoHV-1) are globally important pathogens that significantly affect cattle health and productivity. Both viruses are associated with reproductive disorders, immunosuppression, and increased susceptibility to secondary infections, leading to substantial economic losses in livestock systems [1,2]. Despite their relevance, information on their epidemiology—particularly dual seropositivity dynamics and associated risk factors in dual-purpose cattle systems in Colombia—remains limited.

BVDV, a *Pestivirus* within the family *Flaviviridae*, exhibits high genetic variability and a broad spectrum of clinical and immunological outcomes. The immunomodulatory capacity of BVDV and the latent infection biology of BoHV-1 contribute substantially to viral persistence and herd-level transmission [3,4]. BoHV-1, a member of the *Herpesviridae* family, is a major cause of respiratory and reproductive disease and is characterized by lifelong latency with periodic reactivation [4]. Both viruses play a key role in the bovine respiratory disease complex (BRDC), often interacting with bacterial pathogens [2]. Experimental and clinical studies have shown that concurrent infection with BVDV and BoHV-1 may exacerbate disease severity through synergistic effects on host immune responses [2,3].

The Eastern Plains of Colombia represent a major cattle production region and a key livestock hub characterized by extensive animal movement and generally with low technology adoption [5,6], which may facilitate viral transmission. However, epidemiological data on BVDV and BoHV-1 in this region remain scarce. Therefore, this study aimed to estimate the seroprevalence and dual seropositivity of BVDV and BoHV-1 in dual-purpose cattle in Villavicencio, and to identify epidemiological factors associated with BVDV seropositivity, BoHV-1 seropositivity, and dual seropositivity.

## 2. Materials and Methods

### 2.1. Study Area

**Figure 1:**
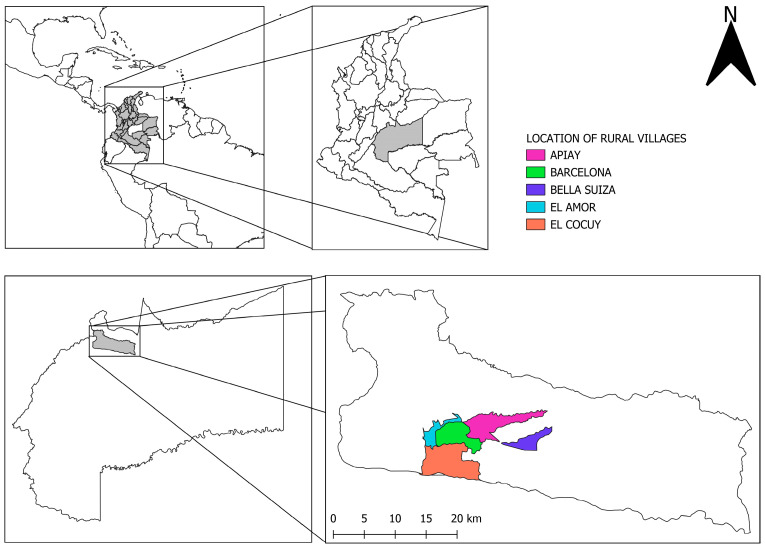
Geographic location of the study area. The map shows Colombia with the Meta department highlighted in gray. Within the department, the municipality of Villavicencio is displayed, with the five selected rural villages (veredas) indicated in color.

The study area is located in the Piedemonte Llanero subregion (Eastern Plains of Colombia), at 4°9′12″ N latitude and 73°39′06″ W longitude. The area has an average altitude of 388 m above sea level, a mean annual temperature of 27 °C, relative humidity of approximately 80%, and an annual precipitation of 3500 mm. The study was conducted in the rural administrative subdivisions locally known as veredas (rural villages) of Apiay, Cocuy, Amor, Bella Suiza, and Barcelona, located in the peri-urban area of the municipality of Villavicencio, Meta, Colombia (Figure 1).

This region represents an important livestock production area in the municipality and a commercial hub with high animal movement, which may facilitate the transmission of infectious diseases. The production system is predominantly dual-purpose (milk and meat), based on extensive grazing under low biosecurity conditions. Additionally, close proximity between farms and frequent animal exchange may contribute to the circulation of viral pathogens in the area.

### 2.2. Study Design

A cross-sectional observational study was conducted between January and June 2017 on cattle farms operating under open production systems. Animals belonged to dual-purpose systems (milk and meat), including predominantly zebu breeds (*Bos indicus*) and their crosses with European breeds (*Bos taurus*). Farms were characterized by low biosecurity conditions.

Sample size estimation was based on a cattle population of 11,971 animals, according to the most recent regional livestock census [7]. The sample size was calculated using a 95% confidence level, an expected prevalence of 30% for BoHV-1 [7], and a precision of 2.7%, using Epidata version 3.1 [8]. A total of 1000 animals from 30 farms across the five study villages were included. At the time of study design, the most reliable prevalence estimate available for the region corresponded to BoHV-1 and was therefore used for sample size calculation. Because reported BVDV prevalence values were generally lower, the resulting sample size was considered sufficient for estimating seroprevalence of both pathogens.

Farms were selected based on accessibility and willingness of farmers to participate. Within each farm, animals were proportionally allocated and randomly selected according to herd size and age groups. The primary sampling unit was the individual animal, with clustering at the farm level. Epidemiological information was collected through a structured questionnaire administered to farm owners/managers. The questionnaire included information on animal movement, reproductive management, feeding practices, biosecurity measures, water sources, and the presence of other domestic animal species. The complete questionnaire is provided as Supplementary Material (Supplementary File S1).

### 2.3. Sample Collection

Blood samples were collected from apparently healthy cattle, regardless of age or sex. From each selected animal, approximately 5.0 mL of blood was obtained via coccygeal venipuncture using vacuum tubes without anticoagulant (Becton Dickinson, Franklin Lakes, NJ, USA).

### 2.4. Laboratory Analysis

Blood samples were labeled and transported at 4 °C to the Laboratory of Genetics and Animal Reproduction at the Universidad de los Llanos (Villavicencio, Colombia). Samples were centrifuged at 2500 rpm for 10 min, and serum was separated using sterile Pasteur pipettes. Serum aliquots (1.0 mL) were stored at −70 °C until analysis. Antibodies against BVDV (p80 protein) were detected using a competitive enzyme-linked immunosorbent assay (ELISA) (INgezim PESTIVIRUS Compac, Ingenasa, Madrid, Spain), which reports 90% sensitivity and 96% specificity. For detection of antibodies against BoHV-1 (gB protein), a blocking ELISA (INgezim IBR Compac, Ingenasa, Madrid, Spain) was used. This kit reports 92% sensitivity and 99% specificity All assays were performed according to the manufacturer’s instructions. Optical density was measured at 450 nm using a microplate spectrophotometer (BioTek EON, Winooski, VT, USA). Samples were interpreted as positive or negative according to the cut-off values provided by the manufacturer.

### 2.5. Data Analysis

Data were recorded and organized in an electronic spreadsheet (Microsoft Excel) prior to analysis. The normality of continuous variables was assessed using the Shapiro–Wilk test. Variables that did not follow a normal distribution were analyzed using non-parametric tests (Kruskal–Wallis). Seroprevalence was calculated as the proportion of ELISA-seropositive animals at the individual level and stratified by farm, sex, and age group. Confidence intervals (95%) for proportions were estimated using the Wilson method [9]. Associations between categorical variables and seroprevalence were evaluated using the chi-square test of independence. A *p*-value ≤ 0.05 was considered statistically significant. Statistical analyses were performed using IBM SPSS Statistics for Windows, version 22.0 (IBM Corp., Armonk, NY, USA).

### 2.6. Multivariate Analysis

To identify independent risk factors associated with seropositivity to BVDV and BoHV-1, multivariate logistic regression models were performed. Additionally, the association between serological responses to BVDV and BoHV-1 was evaluated using chi-square and Spearman correlation analyses. Variables with *p* ≤ 0.20 in univariate analysis were included in the initial model. A backward stepwise selection approach was used. Adjusted odds ratios (OR) and 95% confidence intervals (CI) were calculated. Model fit was evaluated using the Hosmer–Lemeshow test and AIC criteria.

### 2.7. Ethical Considerations

This study was reviewed and approved by the Bioethics Committee of the Universidad de los Llanos (approval No. 08, 2016).

### 2.8. Generative AI Statement

AI-assisted language editing tools were used to check English spelling and grammar and to provide word choice suggestions during manuscript preparation. Additionally, AI tools were used to assist in script code review and graphical generation during data analysis.

## 3. Results

The overall seroprevalence of BVDV, BoHV-1, and BVDV–BoHV-1 dual seropositivity was 17.9%, 55.5%, and 14.0%, respectively. The seroprevalence of BoHV-1 was approximately 3.1 times higher than that of BVDV (Table 1). Geographical location (rural village) was significantly associated with seroprevalence for all three conditions (BVDV: χ^2^ = 30.59, *p* < 0.001; BoHV-1: χ^2^ = 28.32, *p* < 0.001; dual seropositivity: χ^2^ = 17.89, *p* = 0.001) (Table 1). Seroprevalence varied markedly between villages, with BVDV ranging from 10.6% (95% CI: 6.9–15.9) in Barcelona to 32.1% (95% CI: 24.2–41.3) in Apiay, while intermediate values were observed in the remaining villages.

Age group was significantly associated with seroprevalence of BVDV, BoHV-1, and dual seropositivity (BVDV: χ^2^ = 21.59; BoHV-1: χ^2^ = 87.63; dual seropositivity: χ^2^ = 25.44; all *p* < 0.001) (Table 2). For BVDV, a bimodal pattern was observed, with higher prevalence in animals younger than one year (16.8%) and older than three years (21.8%), compared to intermediate age groups. In contrast, BoHV-1 seroprevalence increased progressively with age, reaching the highest values in animals older than three years (67.3%). Dual seropositivity followed a similar age-related pattern, with prevalence increasing from 11.1% in animals younger than one year to 18.2% in animals older than three years. Overall, these age-related patterns differed between viruses.

Sex was significantly associated with BoHV-1 seroprevalence (χ^2^ = 16.43, *p* < 0.001) and dual seropositivity (χ^2^ = 3.87, *p* = 0.049), but not with BVDV (χ^2^ = 0.46, *p* = 0.499) (Table 3). Females showed significantly higher seroprevalence for BoHV-1 (58.7%) compared to males (42.6%), as well as higher dual seropositivity rates (15.1% vs. 9.6%). No significant differences were observed between sexes for BVDV.

At the farm level, seroprevalence varied widely. BVDV seropositivity ranged from 0.0% to 78.7%, BoHV-1 from 0.0% to 100.0%, and dual seropositivity from 0.0% to 52.2%. Approximately 26.7% (8/30) of farms were seronegative for BVDV (95% CI: 14.2–44.5%), whereas only 3.3% (1/30) were seronegative for BoHV-1 (95% CI: 0.6–16.7%). Additionally, 23.3% (7/30) of farms showed no serological evidence of exposure to either virus. The near-universal presence of BoHV-1 at the herd level contrasts with the more heterogeneous distribution of BVDV.

A multivariate logistic regression model was performed to identify independent factors associated with BVDV seropositivity (Table 4). The final model retained five variables after backward stepwise selection: use of artificial insemination, presence of canines, supply of concentrate feed, presence of pigs, and use of surface water sources (streams). The model showed a good overall fit (Omnibus test, *p* = 0.001; Nagelkerke R^2^ = 0.29). The direction and magnitude of the associations were consistent with the regression coefficients (β), confirming the protective or risk effects of the identified variables (Table 4).

Artificial insemination (OR = 0.26; 95% CI: 0.16–0.43), presence of canines (OR = 0.29; 95% CI: 0.17–0.49), and use of concentrate feed (OR = 0.13; 95% CI: 0.04–0.43) were identified as protective factors associated with lower odds of BVDV seropositivity. In contrast, the presence of pigs (OR = 2.19; 95% CI: 1.38–3.48) and the use of surface water sources such as streams (OR = 5.61; 95% CI: 2.85–11.06) were significantly associated with increased odds of seropositivity. Notably, the use of surface water sources showed the strongest association, with more than a fivefold increase in the odds of seropositivity. These findings indicate associations between management practices, environmental factors, and BVDV seropositivity in dual-purpose cattle systems.

A multivariate logistic regression model was also performed to identify factors independently associated with BoHV-1 seropositivity (Table 5). The final model retained four variables after backward stepwise selection: technical assistance, presence of equines, outgoing animal movement, and concentrate feeding. The model showed acceptable fit (Omnibus test, *p* = 0.001; Nagelkerke R^2^ = 0.12).

Technical assistance was identified as a protective factor associated with lower odds of BoHV-1 seropositivity (OR = 0.47; 95% CI: 0.35–0.62). In contrast, the presence of equines (OR = 3.91; 95% CI: 1.69–9.08), outgoing animal movement (OR = 1.76; 95% CI: 1.28–2.42), and concentrate feeding (OR = 2.17) were significantly associated with increased odds of seropositivity. Notably, the presence of equines showed the strongest association with BoHV-1 seropositivity.

A significant association was observed between serological responses to BVDV and BoHV-1 (χ^2^ = 16.75, *p* < 0.001). Among animals seropositive for BVDV, 69.3% were also seropositive for BoHV-1. Spearman correlation analysis showed a weak but significant positive correlation between serological responses to both viruses (r = 0.12, *p* < 0.001), supporting the occurrence of non-random concurrent seropositivity of these pathogens in the study population.

The wide variability observed between farms further highlights the importance of herd-level factors, such as biosecurity practices, animal movement, and management conditions, in shaping infection dynamics.

## 4. Discussion

The results of this study demonstrate evidence of previous exposure to BVDV and BoHV-1 in dual-purpose cattle in Villavicencio, with seroprevalence and dual seropositivity patterns highlighting the epidemiological relevance of these viruses in the region. These pathogens are widely distributed worldwide and are associated with substantial economic losses in cattle production systems due to their impact on reproduction, immunity, and productivity [1].

The seroprevalence of BVDV (17.9%) observed in this study was lower than that reported in other regions of Colombia [10,11,12]. It was also considerably lower than the global prevalence estimated in a meta-analysis including 73 countries (46.23–48.73%; [13]). Rather than indicating stable endemic circulation, this finding likely reflects lower transmission intensity in the study region, potentially associated with reduced animal density, limited introduction of infected animals, or localized management practices. The heterogeneous distribution of BVDV at the farm level further supports this interpretation, suggesting that transmission is not uniform and may depend on specific herd-level factors. The maintenance and spread of BVDV within cattle populations are strongly influenced by the presence of persistently infected animals, which act as major reservoirs and continuous sources of viral transmission within herds [14].

In contrast, the seroprevalence of BoHV-1 (55.5%) was substantially higher and showed a near-universal distribution across farms, consistent with its biological capacity to establish lifelong latency and reactivate under stress conditions. This pattern aligns with previous reports indicating that herpesviruses persist within herds through latency and periodic reactivation [15,16]. The marked difference between BVDV and BoHV-1 at the herd level suggests distinct transmission dynamics, with BoHV-1 being more efficiently maintained within cattle populations. These findings support widespread historical exposure and long-term maintenance of both viruses within cattle populations.

Geographical location was significantly associated with seroprevalence, with higher values observed in Apiay and Cocuy. These areas are characterized by increased animal movement and proximity between farms, which are recognized drivers of infectious disease transmission [17]. Previous studies have demonstrated that cattle movement networks and spatial connectivity play a key role in the spread of BVDV and BoHV-1 [18,19]. The spatial clustering observed in this study supports the hypothesis that local transmission dynamics are influenced by animal movement and farm connectivity.

Age-related patterns revealed important differences between viruses. BoHV-1 seroprevalence increased progressively with age, consistent with cumulative exposure over time and with the well-recognized epidemiological pattern described in both vaccinated and non-vaccinated cattle populations, where the probability of seropositivity increases as animals remain longer within the production system and accumulate opportunities for viral exposure [20]. In contrast, BVDV showed a bimodal distribution, with higher prevalence in animals younger than one year and older than three years. This pattern may reflect early exposure influenced by maternally derived antibodies and age-dependent susceptibility, followed by increased exposure risk in older animals. Similar age-related dynamics have been described for BVDV, in which passive immunity acquired through colostrum and cumulative exposure contribute to shaping serological profiles [21]. Dual seropositivity followed a similar age-related trend, reinforcing the role of cumulative exposure in multi-pathogen dynamics.

Sex-related differences were observed for BoHV-1 and dual seropositivity, with higher prevalence in females. This may be associated with management practices, as females are typically retained longer in herds for reproductive purposes [22], increasing their likelihood of exposure. In contrast, no differences were observed for BVDV, suggesting that transmission of this virus may be less influenced by sex-related management factors.

The prevalence of dual seropositivity (14%) and its distribution across age groups, sex, and geographical areas suggest non-random concurrent seropositivity of both viruses. The significant association between serological responses to BVDV and BoHV-1 further supports the hypothesis that both pathogens share common epidemiological drivers within the study population. Although serological data do not allow confirmation of simultaneous active infection, dual seropositivity may indicate that animals have been exposed to both pathogens during their lifetime. Experimental studies have demonstrated that concurrent infection with BVDV and BoHV-1 can exacerbate respiratory disease, alter host immune responses, and contribute to increased disease severity [23,24,25]. These interactions are particularly relevant within the context of the bovine respiratory disease complex (BRDC), where multiple pathogens may interact to influence clinical outcomes.

The wide variability observed between farms further highlights the importance of herd-level factors, such as biosecurity practices, animal movement, and management conditions, in shaping infection dynamics. Similar findings have been reported in studies evaluating risk factors for BoHV-1 introduction and spread, where management practices and herd connectivity were identified as key determinants [26]. Previous studies have shown that inadequate biosecurity measures and herd management practices contribute significantly to the introduction and maintenance of infectious diseases in cattle populations [27,28].

The multivariate analysis (Table 4) identified several management and environmental factors independently associated with BVDV seropositivity. The use of artificial insemination was associated with reduced odds of infection, which may reflect improved reproductive management [29] and reduced direct animal-to-animal contact compared to natural mating. The protective association observed for artificial insemination is biologically plausible and consistent with previous knowledge of BVDV transmission dynamics. Reproductive management based on artificial insemination reduces direct contact between cows and breeding bulls, thereby limiting opportunities for reproductive transmission and decreasing the risk of introducing infected animals into the herd. In addition, semen used in organized artificial insemination programs is commonly obtained from animals subjected to sanitary monitoring and health certification procedures, which further reduces the likelihood of pathogen dissemination through reproductive practices [30,31]. Beyond the direct effect of insemination itself, the use of artificial insemination may also reflect a higher level of herd management, technical assistance, and biosecurity, factors that collectively contribute to lowering the risk of viral exposure.

Similarly, the use of concentrate feed may be indicative of more intensive management systems with better overall biosecurity practices [32]. Also, the multivariate model for BoHV-1 identified technical assistance as a protective factor, suggesting that improved veterinary oversight and management practices may reduce viral transmission within herds [33,34,35]. In contrast, as reported for other bovine diseases [36], the presence of equines and outgoing animal movement were associated with increased odds of seropositivity, reinforcing the role of animal movement and interspecies interactions in the epidemiology of BoHV-1, however the association between the presence of equines and increased odds of BoHV-1 seropositivity should be interpreted cautiously. Given the strong host specificity of alphaherpesviruses, direct transmission between horses and cattle is unlikely to have epidemiological significance. Instead, the observed relationship probably reflects management practices commonly used in the dual-purpose production systems of the Colombian Eastern Plains. In this region, horses play a central role in daily cattle handling activities, including herding, gathering, sorting, and moving animals across paddocks and grazing areas. Farms that maintain equines often rely on more frequent animal movement and handling, practices that may increase physiological stress in cattle. This observation is particularly relevant for BoHV-1 because, following primary infection, the virus establishes lifelong latency, mainly within sensory ganglia. Reactivation of latent infection can occur in response to stressors such as transport, handling, nutritional challenges, parturition, or corticosteroid release, leading to renewed viral shedding and transmission within the herd [37,38]. Therefore, the presence of equines is unlikely to represent a direct biological risk factor. Rather, it may act as an indirect indicator of management intensity and cattle handling practices that favor stress-mediated reactivation of latent BoHV-1 infections and subsequent viral circulation within the herd. Similar interpretations have been proposed in studies emphasizing the importance of stress-related reactivation in the epidemiology and persistence of bovine herpesvirus infections in cattle populations.

The association observed with concentrate feeding may reflect more intensive production systems, where increased animal density and contact rates could facilitate viral transmission. The opposite direction of the association observed for BVDV and BoHV-1 suggests that concentrate feeding may be acting as a proxy for different management dimensions rather than exerting a direct biological effect on infection risk. Also, the presence of canines was associated with lower seroprevalence, although this finding should be interpreted with caution, as it may reflect confounding factors related to farm management rather than a direct biological effect. Unlike other variables identified in the model, there is no well-established biological mechanism suggesting that dogs directly reduce the risk of BVDV transmission in cattle. Therefore, the observed association is unlikely to represent a direct causal effect and may instead reflect unmeasured management characteristics associated with farms where dogs are present. In many dual-purpose production systems, farm dogs are commonly used for surveillance, livestock management, and protection of facilities. Their presence may therefore be indicative of greater human supervision, more intensive farm management, or improved control of animal movement, factors that could indirectly contribute to lower opportunities for pathogen introduction and dissemination. Consequently, canine presence may function as a proxy variable for management practices that were not fully captured by the epidemiological questionnaire. An additional, although speculative, explanation is that farm dogs may help reduce the presence of scavengers, stray animals, or rodents around feed storage areas and animal facilities. These species can contribute to the mechanical movement of biological material within farms, potentially influencing the circulation of infectious agents. However, no evidence currently supports a direct role of dogs in preventing BVDV transmission. Therefore, this finding should be interpreted as an epidemiological association rather than a causal relationship and should be confirmed in future studies specifically designed to evaluate the influence of farm management characteristics on viral exposure.

In contrast, the presence of pigs and the use of surface water sources such as streams were identified as significant risk factors. The association observed with the presence of pigs may reflect shared environmental and management conditions rather than direct interspecies transmission [39]. Therefore, the presence of pigs may function as an indicator of specific management conditions, increased animal density, or biosecurity deficiencies that indirectly favor pathogen circulation within farms.

The strong association observed between the use of surface water sources and BVDV seropositivity likely reflects increased opportunities for contact among neighboring herds. In the dual-purpose cattle systems of the Colombian Eastern Plains, streams and small watercourses are frequently shared by animals from adjacent farms, creating epidemiological links that may facilitate virus transmission. These locations may also favor direct or indirect contact among animals originating from different herds, particularly in areas with low levels of fencing and biosecurity. Under such conditions, infected or persistently infected animals may contribute to local virus dissemination through secretions and excretions deposited near shared water sources. Therefore, the observed association is likely to reflect increased inter-herd connectivity and animal mixing rather than waterborne transmission itself. Similar observations have been reported in epidemiological studies identifying communal grazing areas, shared resources, and unrestricted animal movement as important contributors to BVDV transmission dynamics [22,40]. These findings highlight the importance of limiting opportunities for inter-herd contact and strengthening biosecurity measures aimed at reducing the introduction and circulation of BVDV within cattle populations.

An interesting finding was the age-related distribution of BVDV seropositivity. While the highest prevalence was observed in animals older than three years, a relatively elevated proportion of seropositive animals was also detected among calves younger than one year of age. The increased seropositivity observed in older animals is consistent with cumulative exposure over time, as animals have more opportunities to encounter the virus throughout their productive life. In contrast, the interpretation of serological responses in young calves requires greater caution. Newborn calves acquire maternal immunoglobulins through colostrum, and antibodies against BVDV may persist for several months after birth. Consequently, some seropositive results detected in animals less than one year old may reflect passive transfer of maternal antibodies rather than active infection or previous exposure of the calf itself. The duration of maternally derived antibodies varies according to colostrum intake, antibody concentration, and individual animal factors, but their presence can influence the interpretation of serological surveys in young populations [41]. Therefore, the bimodal age pattern observed for BVDV should be interpreted carefully, as seropositivity in young animals may represent a combination of maternally derived immunity and early-life exposure, whereas seropositivity in adult cattle more likely reflects cumulative natural exposure to the virus.

From a broader perspective, the relatively lower prevalence observed in this study represents both a potential advantage and a risk. On one hand, it suggests reduced infection pressure and potentially lower economic losses [42]. On the other hand, it indicates a susceptible population in which viral introduction could lead to rapid spread. This underscores the importance of implementing proactive surveillance and biosecurity measures, particularly in regions with high animal movement.

This study has several limitations that should be considered when interpreting the results. The cross-sectional design provides a snapshot of serological status at a single point in time and therefore does not allow causal relationships to be established between the identified risk factors and viral exposure. Consequently, the observed associations should be interpreted as epidemiological relationships rather than direct cause-and-effect mechanisms. In addition, farm recruitment was based on accessibility and willingness of producers to participate, which may have introduced some degree of selection bias. Although the sampled farms represent typical dual-purpose production systems in the study area, caution should be exercised when extrapolating these findings to all cattle populations in the Colombian Eastern Plains. Another important consideration is the availability of vaccination records. Complete vaccination histories were not available for all animals included in the study and, although vaccination against BVDV and BoHV-1 is not routinely implemented in most dual-purpose cattle systems of the region, the possibility that some serological responses were influenced by previous immunization cannot be completely excluded. Related to this, the BoHV-1 ELISA employed in this study detects antibodies against glycoprotein B (gB), and because a complementary strategy based on glycoprotein E (gE) testing was not performed, it was not possible to distinguish antibodies induced by natural infection from those potentially induced by vaccination. Likewise, serological assays provide evidence of previous exposure but do not indicate whether infection was active at the time of sampling. Therefore, seropositivity should be interpreted as evidence of exposure to viral antigens rather than proof of ongoing viral circulation in individual animals [43,44]. Finally, samples were collected in 2017, and changes in management practices, animal movement patterns, biosecurity measures, and disease control strategies may have occurred since then. Nevertheless, these data remain valuable because they provide one of the most comprehensive serological assessments available for dual-purpose cattle systems in the Colombian Eastern Plains and establish an important epidemiological baseline for future surveillance and longitudinal studies.

Finally, although BoHV-1 seroprevalence was considerably higher than that of BVDV, the relatively frequent occurrence of dual seropositivity deserves attention. Animals with antibodies against both viruses may reflect shared transmission routes, common management-related risk factors, or repeated exposure to multiple infectious agents within the same production system. Increasing evidence indicates that mixed infections are a key component of respiratory and reproductive disease syndromes in cattle [45]. While the serological approach used in this study does not allow for confirmation of simultaneous active infection, the significant association between serological responses to BVDV and BoHV-1 suggests that both pathogens circulate within similar epidemiological contexts. Future studies incorporating molecular diagnostics, longitudinal follow-up, and pathogen detection methods will be necessary to better understand the temporal dynamics, biological interactions, and clinical implications of exposure to these viruses in cattle populations.

## 5. Conclusions

This study demonstrates widespread serological evidence of exposure of bovine viral diarrhea virus (BVDV) and bovine herpesvirus type 1 (BoHV-1) in dual-purpose cattle systems in Villavicencio, Colombia, with a high seroprevalence of BoHV-1 and a moderate prevalence of BVDV and dual seropositivity. The marked variability observed across villages and farms highlights the heterogeneous nature of infection dynamics in the region.

Age, sex, and geographical location were significantly associated with seroprevalence patterns, suggesting that both cumulative exposure and management-related factors influence virus transmission. Multivariate analyses identified distinct management and environmental factors associated with seropositivity to BVDV and BoHV-1, highlighting the complex epidemiological interactions underlying viral circulation in dual-purpose cattle systems.

The significant association observed between serological responses to BVDV and BoHV-1 suggests non-random concurrent seropositivity of both pathogens within the study population, reinforcing the epidemiological relevance of dual seropositivity in cattle health and productivity.

Notably, the use of surface water sources and the presence of pigs were associated with increased odds of BVDV seropositivity, whereas artificial insemination, concentrate feeding, and the presence of canines were associated with reduced odds. These findings highlight the importance of improving biosecurity practices, optimizing reproductive management, and considering environmental risk factors to reduce viral transmission.

Overall, the results provide baseline epidemiological evidence to support the design and implementation of targeted surveillance and control strategies for BVDV and BoHV-1 in the Eastern Plains of Colombia. Future studies incorporating longitudinal designs and molecular diagnostics are warranted to better understand infection dynamics and to inform effective disease control programs.

## Figures and Tables

**Table 1:** Seroprevalence of BVDV, BoHV-1 and BVDV–BoHV-1 dual seropositivity by village (Vereda) in dual-purpose cattle from Villavicencio, Colombia. Statistical test (χ^2^): BVDV: χ^2^ = 30.59, df = 4, *p* < 0.001; BoHV-1: χ^2^ = 28.32, df = 4, *p* < 0.001; Dual seropositivity: χ^2^ = 17.89, df = 4, *p* = 0.001. Different superscript letters within columns indicate statistically significant differences (*p* < 0.05). CI: Confidence interval.

Village (Vereda)	BVDV (n/N)	% (95% CI)	BoHV-1 (n/N)	% (95% CI)	Dual Seropositivity (n/N)	% (95% CI)
Apiay	36/112	32.1 ^a^ (24.2–41.3)	69/112	61.6 ^a^ (52.4–70.1)	27/112	24.1 ^a^ (17.1–32.8)
Cocuy	24/83	28.9 ^ab^ (20.3–39.4)	46/83	55.4 ^ab^ (44.7–65.6)	18/83	21.7 ^a^ (14.1–31.7)
Amor	25/147	17.0 ^b^ (11.8–23.9)	65/147	44.2 ^b^ (36.4–52.3)	18/147	12.2 ^b^ (7.8–18.5)
Bella Suiza	75/478	15.7 ^b^ (12.7–19.2)	248/478	51.9 ^ab^ (47.4–56.3)	60/478	12.6 ^b^ (9.9–15.8)
Barcelona	19/180	10.6 ^b^ (6.9–15.9)	127/180	70.6 ^a^ (63.5–76.7)	17/180	9.4 ^b^ (5.9–14.6)
Total	179/1000	17.9 (15.6–20.4)	555/1000	55.5 (52.4–58.5)	140/1000	14.0 (12.0–16.3)

**Table 2:** Seroprevalence of BVDV, BoHV-1 and dual seropositivity by age group in dual-purpose cattle from Villavicencio, Colombia. Statistical test (χ^2^): BVDV: χ^2^ = 21.59, df = 3, *p* < 0.001; BoHV-1: χ^2^ = 87.63, df = 3, *p* < 0.001; Dual seropositivity: χ^2^ = 25.44, df = 3, *p* < 0.001. Different superscript letters within columns indicate statistically significant differences (*p* < 0.05). CI: Confidence interval.

Age Group	BVDV (n/N)	% (95% CI)	BoHV-1 (n/N)	% (95% CI)	Dual Seropositivity (n/N)	% (95% CI)
<1 year	38/226	16.8 ^a^ (12.5–22.2)	89/226	39.4 ^a^ (33.2–45.9)	25/226	11.1 ^a^ (7.6–15.8)
1–2 years	8/106	7.5 ^b^ (3.9–14.2)	31/106	29.2 ^b^ (21.4–38.5)	3/106	2.8 ^b^ (0.9–8.0)
2–3 years	5/81	6.2 ^b^ (2.7–13.6)	40/81	49.4 ^a^ (38.8–60.0)	5/81	6.2 ^ab^ (2.6–13.6)
>3 years	128/587	21.8 ^a^ (18.6–25.3)	395/587	67.3 ^c^ (63.4–70.1)	107/587	18.2 ^c^ (15.3–21.5)
Total	179/1000	17.9 (15.6–20.4)	555/1000	55.5 (52.4–58.5)	140/1000	14.0 (12.0–16.3)

**Table 3:** Seroprevalence of BVDV, BoHV-1 and dual seropositivity by sex in dual-purpose cattle from Villavicencio, Colombia. Statistical test (χ^2^): BVDV: χ^2^ = 0.46, df = 1, *p* = 0.499; BoHV-1: χ^2^ = 16.43, df = 1, *p* < 0.001; Dual seropositivity: χ^2^ = 3.87, df = 1, *p* = 0.049. Different superscript letters within columns indicate statistically significant differences (*p* < 0.05). CI: Confidence interval.

Sex	BVDV (n/N)	% (95% CI)	BoHV-1 (n/N)	% (95% CI)	Dual Seropositivity (n/N)	% (95% CI)
Female	147/803	18.3 ^a^ (15.8–21.1)	471/803	58.7 ^a^ (55.2–62.0)	121/803	15.1 ^a^ (12.7–17.7)
Male	32/197	16.2 ^a^ (11.7–22.0)	84/197	42.6 ^b^ (35.9–49.6)	19/197	9.6 ^b^ (6.3–14.6)
Total	179/1000	17.9 (15.6–20.4)	555/1000	55.5 (52.4–58.5)	140/1000	14.0 (12.0–16.3)

**Table 4:** Multivariate logistic regression model for factors associated with BVDV seropositivity in dual-purpose cattle in Villavicencio, Colombia. OR: Odds ratio; CI: Confidence interval. Reference category for all variables: absence (No). Positive β coefficients indicate increased odds of BVDV seropositivity, while negative coefficients indicate protective effects. Model fit: Omnibus test p = 0.001; Nagelkerke R^2^ = 0.29.

Variable	Category (Reference)	β Coefficient	OR	95% CI	*p*-Value
Intercept	—	−1.685	0.19	—	<0.001
Artificial insemination	Yes (No)	−1.342	0.26	0.16–0.43	<0.001
Presence of canines	Yes (No)	−1.229	0.29	0.17–0.49	<0.001
Concentrate feeding	Yes (No)	−2.077	0.13	0.04–0.43	0.001
Presence of pigs	Yes (No)	0.782	2.19	1.38–3.48	0.001
Surface water source (stream)	Yes (No)	1.725	5.61	2.85–11.06	<0.001

**Table 5:** Multivariate logistic regression model for factors associated with BoHV-1 seropositivity in dual-purpose cattle in Villavicencio, Colombia. OR: Odds ratio; CI: confidence interval. Positive β coefficients indicate increased odds of seropositivity, whereas negative coefficients indicate protective effects. Omnibus test: *p* = 0.001; Nagelkerke R^2^ = 0.12.

Variable	Category (Reference)	β Coefficient	OR	95% CI	*p*-Value
Intercept	—	−1.365	0.26	—	<0.001
Technical assistance	Yes (No)	−0.763	0.47	0.35–0.62	<0.001
Presence of equines	Yes (No)	1.364	3.91	1.69–9.08	0.001
Animal movement (outgoing cattle)	Yes (No)	0.567	1.76	1.28–2.42	<0.001
Concentrate feeding	Yes (No)	0.774	2.17	1.48–3.18	<0.001

## Data Availability

The data supporting the findings of this study are available from the corresponding author upon reasonable request.

## Supplementary Materials

The following supporting information can be downloaded at https://www.mdpi.com/article/10.3390/v18070723/s1: Supplementary File S1: Epidemiological questionnaire used during farm visits.

## Abbreviations

The following abbreviations are used in this manuscript:

BVDV	Bovine viral diarrhea virus
BoHV-1	Bovine herpesvirus type 1
BRDC	Bovine respiratory disease complex
ELISA	Enzyme-linked immunosorbent assay
CI	Confidence interval
OR	Odds ratio
AIC	Akaike information criterion
IBM SPSS	International Business Machines Statistical Package for the Social Sciences
