# Supplementary material for: Seroprevalence, Dual Seropositivity, and Associated Risk Factors of BVDV and BoHV-1 in Dual-Purpose Cattle from the Colombian Eastern Plains
Source: Viruses. 2026 Jun 30;18(7):723. doi: 10.3390/v18070723 (PMC13431480; doi:10.3390/v18070723)
Supplement: Supplementary file 1 [file viruses-18-00723-s001.zip › viruses-4346583-supplementary.pdf]

SUPPLEMENTARY FILE S1. Epidemiological Questionnaire Used During Farm Visits

Section 1. Farm Identification

1. Date of visit: ___________________
2. Farm name: ___________________
3. Municipality: ___________________
4. Village (Vereda): ___________________
5. Geographic coordinates: Latitude: ___________________ Longitude: ___________________
6. Altitude (m.a.s.l.): ___________________
7. Farm area (ha): ___________________
8. Land tenure: □ Owned □ Rented
9. Production system: □ Beef production □ Cow-calf system □ Dual-purpose (milk and beef)
10. Main crops present on the farm:

Section 2. Cattle Population

Number of animals by age and sex category:

**Category**	**Number**
Females <1 year	
Females 1–2 years	
Females 2–3 years	
Females >3 years	
Males <1 year	
Males 1–2 years	
Males 2–3 years	
Males >3 years	

11. Introduction of cattle during the previous six months: □ Yes □ No
12. Removal/sale of cattle during the previous six months: □ Yes □ No

Section 3. Presence of Other Domestic Animal Species

**Species**	**Number**
Swine	
Equines	
Buffaloes	
Backyard poultry	
Fish farming units	
Dogs	

Section 4. Feeding Practices

**Practice**	**Yes**	**No**
Grazing	□	□
Conserved forages	□	□
Agro-industrial by-products	□	□
Concentrate supplementation	□	□

Section 5. Reproductive Management

Practice	Yes	No
Natural mating	◻	◻
Artificial insemination	◻	◻
Embryo transfer	◻	◻

Section 6. Infrastructure and Technical Assistance

Item	Yes	No
Cattle chute	◻	◻
Scale	◻	◻
Corral	◻	◻
Loading ramp	◻	◻
Veterinary medicine cabinet	◻	◻
Electric fencing	◻	◻
Hospital pen	◻	◻
Individual needle use	◻	◻
Professional technical assistance	◻	◻
Pesticide application equipment	◻	◻

Section 7. Preventive Health Measures

Vaccination practices:

Vaccine	Yes	No
Foot-and-mouth disease	◻	◻
Brucellosis	◻	◻
Infectious bovine rhinotracheitis (IBR)	◻	◻
Bovine viral diarrhea (BVD)	◻	◻
Rabies	◻	◻
Other (specify)	◻	◻

Section 8. Reproductive Health Events During the Previous Year

Event	Yes	No
Abortion	◻	◻
Retained placenta	◻	◻
Dystocia	◻	◻
Stillbirths	◻	◻
Mastitis	◻	◻
Diarrhea in adult cattle	◻	◻
Placenta burial/disposal	◻	◻

Section 9. Calf Health Events During the Previous Six Months

Event	Yes	No
Weak calves at birth	□	□
Respiratory disease	□	□
Polyarthritis	□	□
Umbilical disorders	□	□
Hemorrhagic diarrhea	□	□
Other diarrhea	□	□

Section 10. General Practices and Water Sources

Water source available on the farm:

Water source	Yes	No
Community aqueduct	□	□
River	□	□
Stream/creek	□	□
Well	□	□

Rodent control: □ Yes □ No
